# DcR3 binds to ovarian cancer via heparan sulfate proteoglycans and modulates tumor cells response to platinum with corresponding alteration in the expression of BRCA1

**DOI:** 10.1186/1471-2407-12-176

**Published:** 2012-05-14

**Authors:** Joseph P Connor, Mildred Felder, Arvinder Kapur, Nonyem Onujiogu

**Affiliations:** 1 Department of Obstetrics and Gynecology, Division of Gynecologic Oncology, The University of Wisconsin School of Medicine and Public Health, 1212 Sherman Ave, Madison, WI, 53703, USA

## Abstract

**Background:**

Overcoming platinum resistance is a major obstacle in the treatment of Epithelial Ovarian Cancer (EOC). In our previous work Decoy Receptor 3 (DcR3) was found to be related to platinum resistance. The major objective of this work was to define the cellular interaction of DcR3 with EOC and to explore its effects on platinum responsiveness.

**Methods:**

We studied cell lines and primary cultures for the expression of and the cells ability to bind DcR3. Cells were cultured with DcR3 and then exposed to platinum. Cell viability was determined by MTT assay. Finally, the cells molecular response to DcR3 was studied using real time RT-PCR based differential expression arrays, standard RT-PCR, and Western blot.

**Results:**

High DcR3 in the peritoneal cavity of women with EOC is associated with significantly shorter time to first recurrence after platinum based therapy (*p* = 0.02). None-malignant cells contribute DcR3 in the peritoneal cavity. The cell lines studied do not secrete DcR3; however they all bind exogenous DcR3 to their surface implying that they can be effected by DcR3 from other sources. DcR3s protein binding partners are minimally expressed or negative, however, all cells expressed the DcR3 binding Heparan Sulfate Proteoglycans (HSPGs) Syndecans-2, and CD44v3. DcR3 binding was inhibited by heparin and heparinase. After DcR3 exposure both SKOV-3 and OVCAR-3 became more resistant to platinum with 15% more cells surviving at high doses. On the contrary CaOV3 became more sensitive to platinum with 20–25% more cell death. PCR array analysis showed increase expression of BRCA1 mRNA in SKOV-3 and OVCAR-3 and decreased BRCA1 expression in CaOV-3 after exposure to DcR3. This was confirmed by gene specific real time PCR and Western blot analysis.

**Conclusions:**

Non-malignant cells contribute to the high levels of DcR3 in ovarian cancer. DcR3 binds readily to EOC cells via HSPGs and alter their responsiveness to platinum chemotherapy. The paradoxical responses seen were related to the expression pattern of HSPGs available on the cells surface to interact with. Although the mechanism behind this is not completely known alterations in DNA repair pathways including the expression of BRCA1 appear to be involved.

## Background

**Figure 1:**
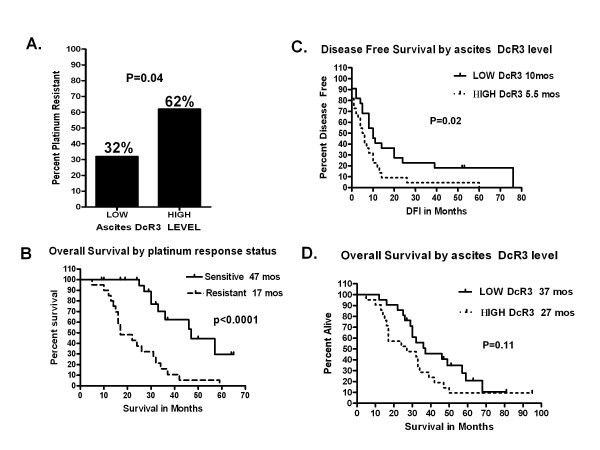
**HIGH ascites levels of DcR3 are associated with platinum resistance in women with EOC.** Ascites from forty five women with stage IIIC-IVA ovarian cancer were tested for DcR3 by ELISA and the cohort divided at the median level into HIGH and LOW DcR3 groups. **A**. Women with HIGH DcR3 were almost twice as likely to have platinum resistant disease. **B**. As would be expected women in this population with platinum resistant disease had significantly shorter overall survival (see ref. 8). **C**. Women with HIGH DcR3 levels had a significantly shorter time to first recurrence after primary therapy and a trend (**D**) towards shorter overall survival.

DcR3, also known as TR6, M68, or TNFRSF6B is a soluble protein member of the tumor necrosis factor receptor family. DcR3 is known to prevent apoptosis via direct ligand binding of Fas ligand, LIGHT and TL1A, acting as a decoy for their intended death receptor, Fas, HVEM/LTβR, and DR3 respectively [1,2]. DcR3 has been identified in tumor tissue and has been shown to be elevated in the serum of cancer patients were its expression is often predictive of poor survival [3-7]. We have previously reported the presence of functional DcR3 in advanced Epithelial Ovarian Cancer (EOC) ovarian cancer demonstrating that naturally occurring DcR3 inhibited Fas-ligand mediated apoptosis. DcR3 was found to be concentrated in ascites fluid in all cases of advanced stage disease and higher levels in the peritoneal cavity were associated with platinum resistant cases. In this cohort, women with high (greater than the median level) ascites DcR3 levels were almost twice as likely to manifest platinum resistant disease compared to women with low levels (62 vs 32% platinum resistant disease (Figure 1A))[8]. 

Despite advances in surgical care and improved chemotherapeutic agents EOC remains the most lethal of gynecologic malignancies. It is estimated that 23–25,000 US women are affected annually and unfortunately the majority of them will die of their disease. Aggressive cytoreductive surgery followed by platinum based chemotherapy is the mainstay of therapy for these women yet approximately 20% of women treated this way will not respond to this therapy and are considered platinum refractory. Equally discouraging, another 10- 20% will be identified with recurrent disease less than 6 months after the completion of platinum based therapy, bringing the total to 30-40% of women having platinum resistant disease [9]. Unfortunately once disease has recurred the opportunity for curative therapy is considered lost. Since platinum is the cornerstone of ovarian cancer treatment and platinum resistance results in incurable disease an improved understanding of the mechanisms of resistance could have major impact on the management of this disease. To better understand the association of DcR3 and platinum resistance we explored the role of DcR3 in the response of ovarian cancer cells lines to platinum.

## Methods

### Antibodies and other reagents

Fas (CD95) and DR3 antibodies were purchased from eBioscience. Fas ligand antibody was from BD Biosciences and the BRCA1 antibody (Ab1) was from Calbiochem/EMD chemicals. LIGHT (CD258), LTβR, HVEM, TL1A, CD44v3, Syndecan-2 antibodies as well as rhDcR3-Fc, rhIgG-Fc, and the DcR3 ELISA reagents were obtained from R&D Systems. The source for heparin and heparinase 1 was Sigma-Aldrich. FITC-conjugated goat anti-human Fc fragment was purchased from Jackson ImmunoResearch Laboratories. The cancer cell lines SKOV-3, OVCAR-3, CaOV-3, SW626, and SW480 were obtained from ATCC. The EOC cell line A2780 and its platinum resistant sub-clones were provided by Dr. Thomas Hamilton, Fox Chase Cancer Institute.

### Human subjects and clinical samples

All women taken to the operating room by the gynecologic oncology service at the University of Wisconsin with a presumed diagnosis of EOC were asked to participate in this IRB approved protocol. Informed consent was obtained for the collection of ascites fluid and tumor tissue at the time of cytoreductive surgery. Ascites was centrifuged at 1200 rpm for 20 min and the supernatant fluid frozen at −20°C until assayed by ELISA. The cellular fraction was frozen in liquid nitrogen in fetal calf serum with DMSO until thawed for primary cultures. Demographic information and survival data was taken from the medical record. Disease free and overall survivals were compared by the method of Kaplan Meier with the significance of differences determined by Log rank test. In all analysis significance was set at *P* ≤ 0.05. A second IRB approved protocol was used for the collection of residual ascites fluid from subjects with no known clinical history of cancer that were undergoing therapeutic paracentesis. This fluid was processed for ELISA and primary cell culture as stated above for EOC samples.

### Primary cultures

Cell fractions were thawed, washed in sterile PBS and seeded in 25 cm^2^ flasks in RPMI media with 10% fetal calf serum at a cell density of 1X10^6^ cells/flask. For adhesion based tumor enrichment experiments the cells were allowed to attach at 37°C for one hour then all none adherent cells were transferred to a new flask and allowed to attach for 24 h. All cultures were washed and fresh media added after 24 h. Media samples for DcR3 were then collected at 72 h and frozen at −20°C until ELISA for DcR3 was done. Cells were analyzed for EpCAM expression by flow cytometry at the time of media collection.

### Flow cytometry

The expression the protein binding partners of DcR3 and their native ligands (Fas, FasL, LIGHT, LTβR, HVEM, DR3, and TL1A) was determined by flow cytometry. The listed antibodies were incubated with cells for 30 min at 4°C using manufacturer-recommended concentrations. Cells were then washed and incubated with 1.5 μg of streptavidin PE (if necessary) for a further 30 min at 4°C. After secondary incubation, cells were washed and analyzed on the cytometer, BD Biosciences FACSCalibur. All flow experiments were performed with the addition of propidium iodide to enable the exclusion of dead cells from the analysis. Flow data analysis was performed with Treestar’s FloJo software.

To determine the ability of cell lines to bind DcR3 cells were detached using Puck’s EDTA, then divided into the appropriate tubes and incubated with either 1 μg/200μL of rhDcR3-Fc or rhIgG-Fc for 45 min at 4°C. Cells were then washed in 1% FBS-PBS and further incubated with 1.5 μg of goat anti-human Fc FITC for 30 min at 4°C. Cells were washed again and fluorescence signal of the stained cells was detected using a BD Biosciences FACSCalibur. Initial experiments showed no difference between cells stained with rhIgG-Fc + anti-human Fc FITC and anti-Fc FITC alone, so anti-Fc alone was used as the negative control.

In order to block the rhDcR3-Fc from binding 1 μg of rhDcR3-Fc or rhIgG-Fc was mixed with 500U of heparin and incubated for 30 min at room temperature before adding to the cells for staining as described above.

In experiments requiring the removal of heparin binding sites prior to incubation with rhDcR3-Fc, cells were detached as before, then incubated with 20U/mL heparinase or 0.25% trypsin for 2 h at 37°C with periodic agitation. Cells were then washed and stained as stated previously.

### Platinum cytotoxicity assays

A single flask each of SKOV-3, OVCAR-3 and CaOV-3 cells were split equally by trypsin EDTA. The media for one daughter flask from each cell line was supplemented with rhDcR3-Fc at a concentration of 0.1 μg/ml. The DcR3 treated cells were then maintained in media with continued DcR3 treatment and passed along side of the untreated control cells (from the same original culture) for 12 weeks. At this point cells from each of the 6 cell lines were expanded and frozen for future use. Since we had theorized that DcR3 would result in increased platinum resistance we elected to treat each cell line with a range of high dose platinum to test this theory. Cells were harvested and plated in 24 well plates in triplicate. After allowing 24 h for cell attachment, media was replaced and wells were treated in triplicate with cis-platinum at final concentrations of 2.5-25 μg/ml. After 72 h cell viability was determined by MTT assay (Sigma-Aldrich) and platinum response was reported as percent of untreated control wells. ANOVA with Bonferroni’s multiple comparison was used to assess for differences based on DcR3 treatment and between platinum doses. These experiments were repeated four times over a series of passages.

### Real time RT-PCR array

SKOV-3, CaOV-3 and OVCAR-3 cells were grown for three months either untreated or continuously treated with 0.1 μg/ml rhDcR3-Fc. The cells were homogenized in Trizol at 80-90% confluence and RNA was extracted according to the manufacturer’s instructions (Sigma, St Louis). Extracted RNA was further purified using RNeasy kit (Qiagen) according to the manufacturer’s protocol. RNA purity was assessed using Nano-drop spectrophotometer, which showed the 260/280 ratio of all the samples were 2.0 or greater. Purified RNA was reverse transcribed to make cDNA using SABiosciences first strand cDNA synthesis kit (C-033). The changes in gene expression of treated cells compared to untreated cells were determined by analyzing the cDNA on the 96 well Cancer Pathway Finder PCR array from SABiosciences (PAHS-033) on a Bio-Rad iCycler, using RT^2^ real time SYBR green PCR master mix PA-011. Genes of interest were defined as any gene with at least 1.5 fold change in the same direction in at least two of three replicate samples.

### Real time PCR for BRCA-1

The untreated and DcR3 treated cells (SKOV-3, CaOV-3 and OVCAR-3) were homogenized in Trizol (Sigma, Cat No T9424) and RNA was extracted according to the manufacturer’s instructions. The RNA was reverse transcribed into cDNA using Omniscript RT kit from Qiagen (Cat. No.205111). The BRCA-1 and S27 were amplified with the primers obtained from SA Biosciences (Real time PCR primers for Human BRCA1, Cat No. PPH00322E and Human S27, cat No PPH17248B) using SsoFast Evagreen Supermix from BioRad (Cat. No. 172–5201) on CFX96 Real-Time PCR Detection System. The three step cycling conditions used were, 95°C for 30s, followed by 40 cycles of the denaturation at 95°C for 1 s, annealing at 60°C for 5 s. The data was analyzed using the CFX software manager.

### Standard RT-PCR for BRCA-1

The untreated and DcR3 treated cells (SKOV-3, CaOV-3 and OVCAR-3) were homogenized in Trizol and RNA was extracted according to the manufacturer’s instructions. The RNA was reverse transcribed into cDNA using Omniscript RT kit from Qiagen (Cat. No.205111). The BRCA-1 was amplified with the primers (sense, 5’- TGAGGCATCAGTCTGAAAGTTCTGGA-3’ and anti-sense, 5’- CTGATGTGCTTTGTTCTGGA-3’) using fast cycling PCR kit from Qiagen (Cat. No. 203745). The cycling conditions used were, initial activation at 95°C for 5 min, followed by 30 cycles of the denaturation at 96°C for 5 s, annealing at 60°C for 5 s and extension at 68°C for 10s, with final extension at 72°C for 1 min. The PCR product was run on 2.5% agarose gel at 100 V for 2 h and bands were visualized using Flourchem8900 ultraviolet transilluminator. Bands were quantified using Image J (NIH) normalized to GAPDH.

### Western blot analysis for BRCA-1

Ovarian cancer cell lines SKOV-3, CaOV-3 and OVCAR-3 were grown for three months either untreated or treated with DcR3 as described. When the cells were 80-90% confluent, the cells were washed with ice cold PBS and lysed in RIPA lysis buffer containing protease inhibitors. The cell lysate was briefly sonicated and centrifuged then the protein concentration was determined using the BCA method. The protein samples were heat denatured (95°C, 5 min) in Laemmli buffer and proteins (50ug/lane) were separated on 4-15% SDS-PAGE gels, electro blotted on PVDF membrane and probed with a mouse monoclonal antibody (1:50) against human BRCA-1 (clone Ab-1). The membrane was incubated in the primary antibody overnight at 4°C, then washed with PBST before incubating in HRP conjugated goat anti-mouse secondary antibody for 1 h at RT. The membrane was washed again before visualizing the protein with the super signal west femto maximum sensitivity substrate (Pierce). The immunoreactive signals were quantified using Image J (NIH), normalized to β-Actin.

## Results

After additional follow-up, our original cohort of women [8] continue to support a relationship between DcR3 and platinum resistant disease in that women with high ascites DcR3 have a significantly shorter time to first recurrence after platinum based primary therapy, 5.5 months vs. 10 months *p* = 0.02, (Figure 1C). The high DcR3 group also showed a trend towards worse overall survival by ten months (*p* = 0.11, Figure 1D).

**Figure 2:**
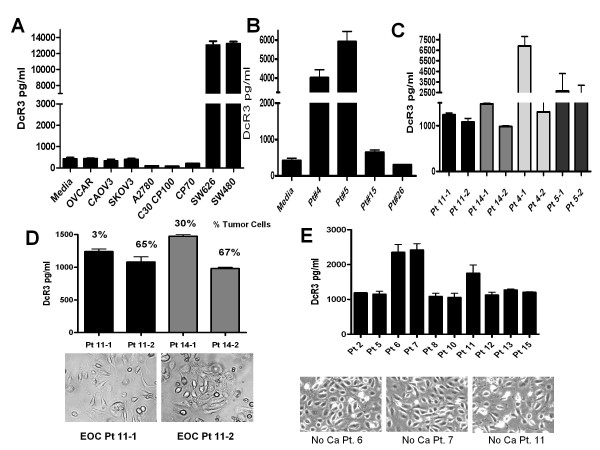
**None-malignant cells contribute to DcR3 in EOC.** DcR3 levels were determined by ELISA and values compared to media devoid of cells. **A**. DcR3 is not produced by the EOC cell lines tested as compared to the colon cancer cell lines SW626 and SW480. **B**. DcR3 is produced by primary culture of peritoneal cells (mix of cells including immune cells, stromal cells, mesothelial cells, and tumor cells) from women with ovarian cancer and the level in culture correlates to the levels in the patient’s ascites fluid (data not shown). **C**-**D**. Cells that adhere to plastic in one hour or less (Pt#-1) produce as much or more DcR3 than cells that were allowed 24 h to adhere (Pt#-2). **D**. Rapidly adherent cells produce as much or more DcR3 however have 50-95% fewer cancer cells than the 24 h samples. **E**. Primary culture of peritoneal cells from patients with none-malignant ascites produce DcR3 (DcR3 is also present in the ascites fluid at levels similar to the malignant ascites of ovarian cancer, data not shown). Taken together these results suggest that non-malignant cells contribute to the DcR3 levels seen in the peritoneal cavity in advanced EOC.

DcR3 is known to be produced by several tumor cell lines including the colon cancer cell lines SW480 and SW626. Contrary to this none of the ovarian cancer cell lines we have tested to date have demonstrated detectable levels of DcR3 in their media (Figure 2A). Of interest, primary cultures of peritoneal cells from women with EOC did secrete DcR3 (Figure 2B). Since these cultures are a mix of tumor cells, mesothelial cells, fibroblasts and immune cells we performed adhesion based cell enrichments for tumor cells (EpCAM positive cells) and found that cells that rapidly adhere to plastic (few tumor cells) produce more DcR3 than the cells from the same sample that were allowed 24 h to attach (enriched for tumor cells, Figure 2C). Flow cytometry for EpCAM as an indicator of tumor cells in these culture pairs found that the second cultures were significantly enriched for tumor cells (60-80% vs. 2-30%) yet this culture produces less DcR3 than the first adhesion culture with only a fraction the number of tumor cells, (Figure 2D). Lastly we cultured peritoneal cells from patients without cancer but who had ascites secondary to chronic liver disease. As seen in Figure 2E these non-malignant peritoneal cells produce DcR3 at levels very similar to the peritoneal cells from ascites of women with ovarian cancer. Taken together these results suggested that cells other than or in addition to tumor cells are a significant source of DcR3 in the peritoneal cavity of women with ovarian cancer. Based on this we theorized that even in cases were the cancer cells do not produce excessive DcR3 (like the ovarian cancer cells lines tested here) they may be influenced by DcR3 produced by other cells in the tumor micro-environment.

**Figure 3:**
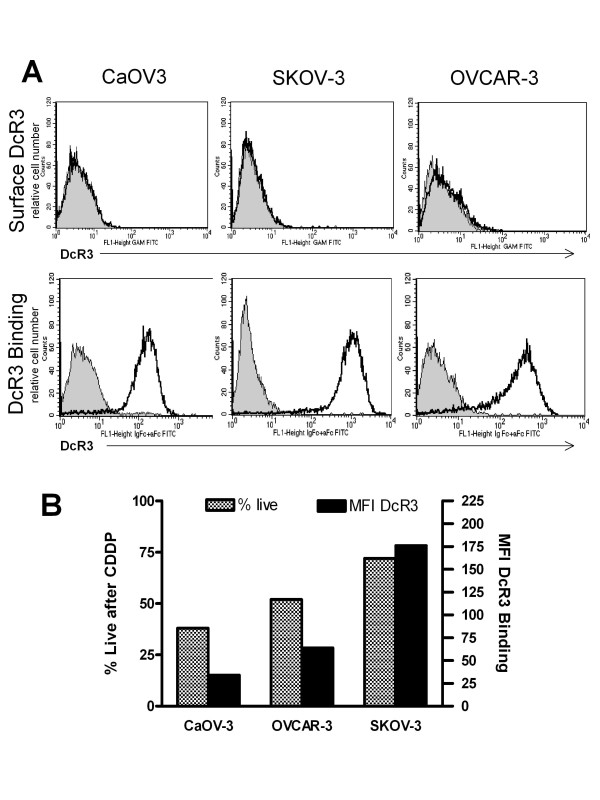
**Ovarian cancer cell lines bind DcR3. A**. Flow cytometry shows that there is no baseline cell surface DcR3 on the EOC cell lines tested (upper row) however recombinant human DcR3 (rhDcR3-Fc, R&D Systems) binds to the surface of EOC cell lines (lower row). Incubated with 1 μg rhDcR3-Fc, SKOV-3 binds the most DcR3 and CaOV-3 binds the least DcR3. **B**, DcR3 binding, defined as MFI by flow cytometry (solid black bars) is in direct proportion to the cell lines sensitivity to platinum (grey hatched bars) consistent with the clinical association of DcR3 to platinum resistant disease (CDDP = Cis-platinum 2.5 μg/ml).

None of the cell lines had evidence of surface bound DcR3 by flow cytometry (Figure 3A, top row), however, all three lines were found to diffusely bind exogenous DcR3 (rhDcR3-Fc) to their cell surfaces. As shown in Figure 3A, bottom row, SKOV-3 cells bind more DcR3 than OVCAR-3 cells and CaOV-3 cells bind the least. We next tested the three cell lines for their sensitivity to platinum and found that in 2.5 μg/ml of cis-platinum (CDDP), CaOV3 was the most sensitive line, SKOV3 was the most resistant, and OVCAR-3 demonstrated an intermediate response to platinum (Figure 3B hatched bars). The cells’ responses to platinum were found to be in direct proportion to the individual cell lines’ degree of DcR3 binding by flow cytometry (Figure 3B solid black bars), i.e. SKOV3 bound the most DcR3 and CaOV3 the least.

**Figure 4:**
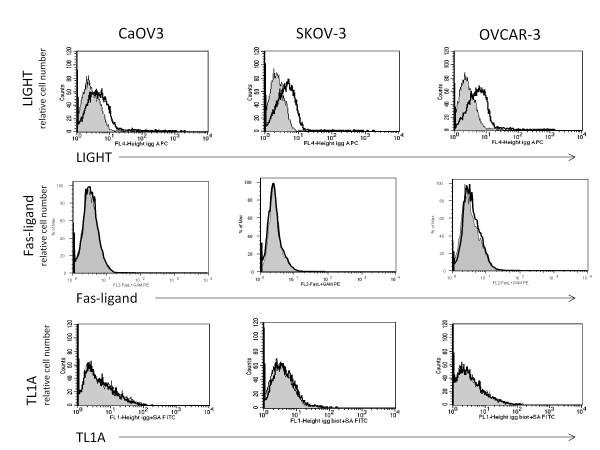
**Ovarian cancer cell lines do not express high levels of DcR3 protein binding partners.** The cell lines studied do not express the protein ligands for DcR3 (except for low levels of cell surface LIGHT) to account for the binding pattern seen in Figure 3A.

To determine the mechanism of binding, we evaluated the cell lines by flow cytometry for the surface expression of the cell surface protein ligands of DcR3: LIGHT, Fas-ligand and TL1A. As seen in Figure 4, although there is a slight positive shift for LIGHT, overall the three protein ligands are not present to a degree that would explain the exogenous DcR3 binding seen in Figure 3A. All of the cell lines tested are known to express surface Fas and the flow cytometry confirms this as well as showing that the cells do not express the other death receptors (HVEM/LTβR and DR3) known to interact with DcR3s protein ligands (data not shown).

**Figure 5:**
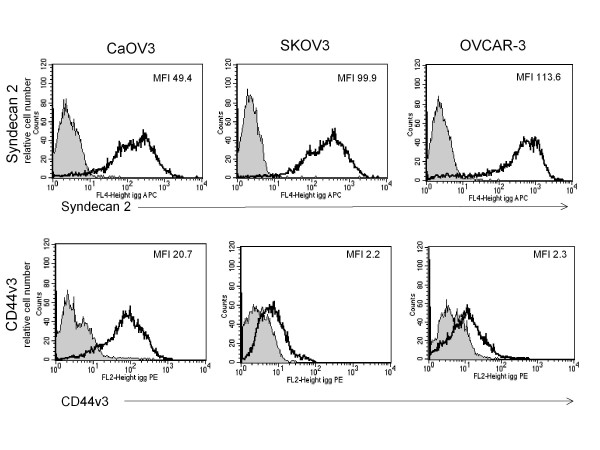
**Ovarian cancer cells express cell surface Heparan Sulfate Proteoglycans known to bind DcR3.** Flow cytometry demonstrates the presence of the known DcR3 binding HSPGs, Syndecan-2 and CD44v3 on the surface of EOC cell lines. Syndecan-2 is the dominant HSPG in SKOV-3 and OVCAR-3 cells where in CaOV-3 Syndecan-2 expression is half that of the other two cell lines but CD44v3 expression is tenfold higher than the other lines.

**Figure 6:**
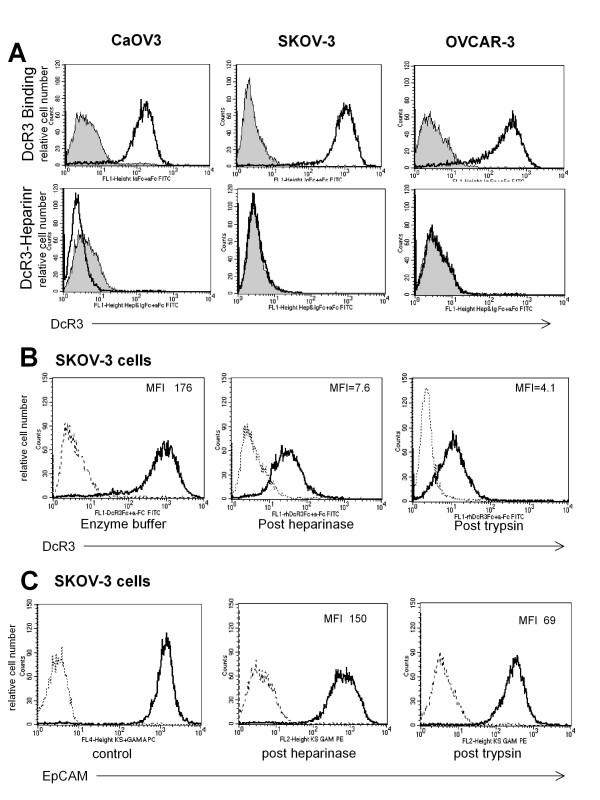
**DcR3 binding to EOC is heparin sulfate dependent. A**. DcR3 binding is completely inhibited by heparin sulfate in all three cell lines. **B**. DcR3 binding is also inhibited by the removal of heparan sulfates from HSPGs on the cells surface with heparinase or by removal of complete HSPGs with their peptide backbone by trypsin. **C**. The surface adhesion molecule EpCAM was used as a control for heparinase and trypsin treatments with appropriate responses seen.

In addition to its anti-apoptotic properties, DcR3 has more recently been found to modulate cellular events independent of its three protein ligands. Based on the presence of a heparin binding motif in the amino acid structure of DcR3, many effects of DcR3 have been found to be mediated via binding to cell surface Heparan Sulfate Proteoglycans (HSPGs)[10,11]. In cells of the immune system the responsible HSPGs are primarily Syndecan-2 and CD44v3 [12,13]. As seen in Figure 5, all three cell lines expressed both Syndecan-2 and CD44v3. In both SKOV-3 and OVCAR-3 there were higher levels of Syndecan-2 (MFI =100-115) as compared to CD44v3 (MFI = 2). Conversely, in CaOV-3 cells there was less Syndecan-2 (MFI = 50) by at least half and 10 fold more CD44v3 (MFI 20.7) than in the other two cell lines. The binding of DcR3 to all three cell lines was completely inhibited by the addition of heparin sulfate to DcR3 prior to its incubation with the cells (Figure 6A). DcR3 binding was also significantly reduced after treatment of the cells with heparinase to remove the heparan sulfate moieties from HSPGs or with trypsin to strip the protein backbone of the HSPGs (Figure 6B). As a flow cytometry control, these treatments appropriately reduced, trypsin, or had no effect, heparinase, on the surface expression of the protein adhesion molecule EpCAM (Figure 6C). Taken together, this indicates that DcR3 interacts with EOC cells via HSPGs. 

**Figure 7:**
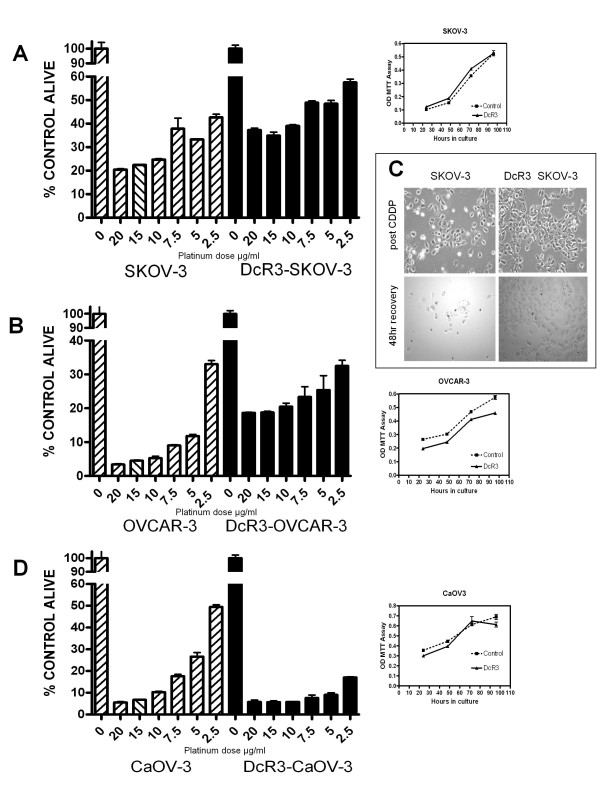
**DcR3 modulates response to platinum in ovarian cancer cell lines. A** and **B,** Chronic exposure to DcR3 in culture (12 weeks) resulted in increased resistance to cis-platinum in SKOV-3 and OVCAR-3 cells with a mean of 15% more viable cells across a wide range of very high platinum doses, *p* < 0.001 by ANOVA. **C**. Photomicrographs demonstrate that DcR3 exposed SKOV-3 cells remain adherent and alive 48 h after platinum has been removed from the cultures where cell numbers continue to decline after platinum has been removed from the parent SKOV-3 cell line. DcR3 treated cells were 60% viable at 48 h by eosin stain. **D**. CaOV-3 cells exposed to DcR3 under identical conditions demonstrated increased cytotoxicity in response to platinum with > 25% more cell death even at the lower doses, *p* < 0.001 by ANOVA. Insets indicate that DcR3 exposure has no significant effects on the growth rate of the cell lines tested.

**Figure 8:**
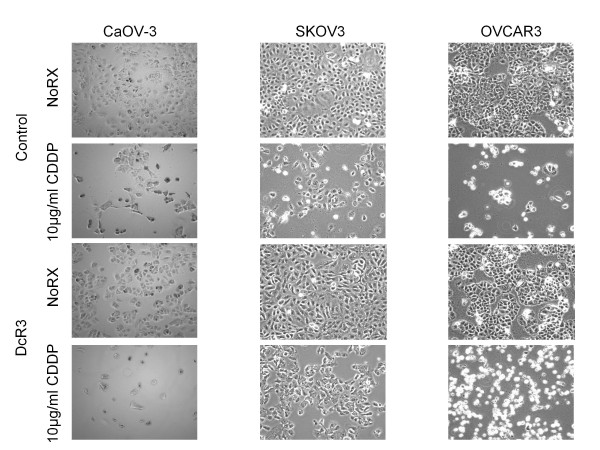
**DcR3 modulates response to platinum in ovarian cancer cell lines.** Representative photomicrographs highlight the paradoxical response to platinum after DcR3 exposure.

Based on our theory that cancer cells can be effected by DcR3 even when they do not over produce DcR3 we chronically cultured each of the 3 EOC cell lines in continuous DcR3 at 0.1 μg/ml for 12 weeks (approximately 20 passages) to determine if there were any effects on cell proliferation or response to platinum. All 3 cell lines became more adherent as evident by an increase in the time needed to harvest the cells in 0.25% trypsin/EDTA solution (data not shown). There were no significant effects on cell proliferation seen in response to DcR3 (Figure 7 growth curve insets for each cell line). Since we were theorizing increased platinum resistance in response to DcR3, after 12 weeks cells were plated and treated with high dose cis-platinum (range 2.5- 20 μg/ml) for 72 h. As seen in Figure 7A-B SKOV3 and OVCAR-3 cells became significantly more resistant to platinum even at the extremely high doses of 15–20 μg/ml, with a mean of 15% more viable cells after 72 h (*p* < 0.001 by ANOVA for both cell lines). This difference became more profound after removal of platinum and continued culture in fresh media. Figure 7C demonstrates that after an additional 48 h in culture the SKOV-3 parent cell line (no DcR3 treatment) showed continued loss of cells while the DcR3 treated cultures remain relatively stable in cell density. Eosin staining of cells demonstrated that 50-60% of these cells were viable. Unexpectedly, the effects on CaOV-3 were the exact opposite as the cells become more sensitive to platinum with 20-25% more cell death (Figure 7D) even at the lowest doses tested. These experiments were repeated a total of 4 times with similar results each time as seen in the representative photomicrographs in Figure 8. 

**Figure 9:**
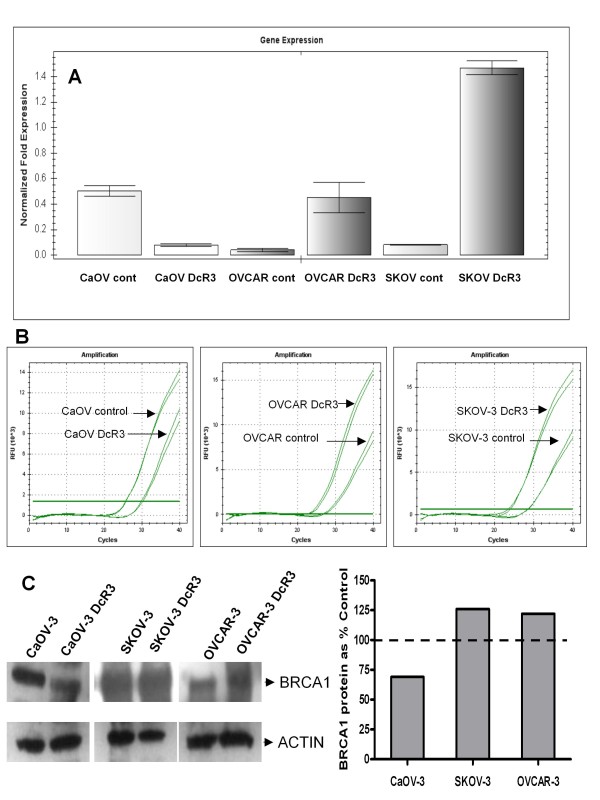
**BRCA1 mRNA and protein expression are modulated by DcR3**. Real time reverse transcriptase PCR (**A**. means of triplicate samples by RT-PCR and **B**. examples of individual melt curves) and Western Blot (**C**) for BRCA1 demonstrate that the differential changes in BRCA1 expression between SKOV-3/OVCAR-3 and CaOV-3 cells exposed to DcR3 are in line with the changes seen in platinum responsiveness from the same DcR3 treated cultures.

This data would suggest that DcR3 can either increase resistance to, or enhance the cytotoxic effects of platinum. To determine if there are any molecular/genetic differences in the effects of DcR3 in each cell line we performed focused real time reverse transcriptase PCR based arrays using the SABiosciences’ (Qiagen) Cancer Pathway Finder system. This 96 well based RT-PCR assay was used to compare the differential expression of 86 cancer associated genes between each cell line and it’s chronically DcR3 treated sub-clone. As seen in table 1, among the three cell lines 55 genes were found to be altered (by at least 1.5 fold change up or down in at least 2 of 3 replicate arrays) after chronic exposure to DcR3. The array data was then analyzed for patterns of gene expression that fit the paradoxical alterations in platinum response after DcR3 exposure between SKOV-3 and OVCAR-3 compared to CaOV3. This resulted in the identification of the 5 genes in table 2. Of these, the pattern seen for BRCA1 mRNA was of particular interest given the known association between BRCA1 expression and response to DNA damaging agents such as platinum. To further assess this pattern EOC cells and their chronically DcR3 exposed variants were tested by standard real time reverse transcriptase PCR and Western Blotting for BRCA1 at the mRNA and protein levels respectively. As seen in Figure 9A-B (A, mean of triplicate samples and B, examples of individual melt curves), RT-PCR confirmed the array results in that BRCA1 mRNA was reduced in CaOV3 cells and increased in SKOV-3 and OVCAR-3 cells after chronic exposure to DcR3 (The same results were seen by standard reverse transcriptase PCR and densitometry for each sample, data not shown). Similar changes were seen at the protein level where BRCA1 was reduced by 31% in CaOV-3 cells and increased by 25% in SKOV-3 and OVCAR-3 cells after chronic DcR3 treatment (Figure 9C).

**Table 1:** Genes altered by chronic DcR3 exposure by real time RT-PCR array

	**SKOV-3**	**OVCAR-3**	**CaOV3**
UP	BRCA1, CDC25A,Chk2,BAD, GZMA, DR3, Fas, ITGA4,B1and B3,MTSS1, SYK, ANGPT1, IL8, PDGFB, TEK, TGFB1, THBS1, MET, MMP1, MTA2,NME1, NME4.PLAU,PLAUR, SERPINE1	BRCA1, CCNE1, S100A4, GZMA, HTATIP2, Fas, FOS, MAP2K1/MEK, NFKB1A, SNGC, ANGPT2, INF-A, INF-B, PDGFA, TNF, MMP2, SERPINE1, TIMP3	CDKN1A (P21), S100A4, TERT, DR3, ITGB3, COL18A1, INF-B, PDGFA, TEK, TGFB1, VEGFA, MMP1, PLAU, SERPINE1, TIMP1
DOWN	ATM, CDKN2A (P16), E2F1, APAF1, BCL2, FOS, ITGB5, ANGPT2, COL18A1, FGFR2, INF-A, INF-B, TGFBR1, TNF, VEGFA, MMP2, MMP9	CHK2, E2F1, TP53, DR3, PIK3R1, ETS2, ITGA4,B3, MCAM,MTSS1, ANGPT1, COL18A1, THBS1, MMP1, MMP9, MTA2, PLAU, SERPINB5, TWIST1	BRCA1, CCNE1, CDK2,CDK4,CHK2, MDM2, DR5, PIK3R1, SNGC, INTGB1, ANGPT1, ANGPT2, FGFR2, PDGFB, THBS1, MET, TIMP3, TWIST1

**Table 2:** **Genes with patterns of altered expression in response to chronic DcR3 that correspond to the alterations in response to platinum as seen in Figure **6

**GENE**	**SKOV3**	**OVCAR3**	**CAOV3**
**BRCA1**	UP	UP	DOWN
**E2F1**	DOWN	DOWN	UP
**GZMA**	UP	UP	N/A
**Fas**	UP	UP	N/A
**COL18A1**	DOWN	DOWN	UP

## Discussion

The overexpression of DcR3 in human malignancies has been show to permit and or promote tumor growth via multiple mechanisms. Many of these have focused on DcR3 effects on the immune system. DcR3s has been reported to effect both T-cell function and chemotaxis as well as to alter local chemokine production to result in a Th2 inflammatory local environment [14-16]. DcR3 also demonstrates diverse effects on cells of monocyte/macrophage lineage were it has been shown to increase monocyte adhesion [13,17], skew macrophage to an M2 tumor associated phenotype, negatively alter their antigen presenting function, and to directly result in the apoptosis of dendritic cells [12,18-22]. Our paper provides data to support a novel pro-tumor effect of DcR3 via its association with platinum resistant disease. Although DcR3 has been reported to be elevated in both the blood and ascites of women with ovarian cancer the functional or prognostic significance of this is not clear [8,23]. Our prior data indicated an association with platinum refractory disease, a finding further supported by the data presented here. Continued follow up has shown a significantly shorter time to first recurrence in women with high peritoneal DcR3 levels confirming our prior association with platinum resistance.

In many cancers studied (colon, pancreas, hepatocellular) DcR3 has clearly been shown to be over produced by cancer cells and there are representative DcR3 producing cancer cell lines. We have seen high levels of DcR3 in the peritoneal cavity of women with advanced EOC yet have not found an EOC cell line that secretes DcR3 into its culture media. This led us to evaluate whether none-malignant cells in the peritoneal cavity from EOC cases were responsible for the DcR3 production in these cancers. This theory is supported by a significant body of evidence for abnormal DcR3 production from many none-malignant cell types including fibroblasts, synovial cells, inflamed GI mucosa, and various cells of the immune system. Although the data presented here does not exclude tumor cells as a partial source of peritoneal DcR3 in EOC we have demonstrated significant production of DcR3 by none-malignant (EpCAM negative) cells and this finding was the rationale for the cell line experiments and interesting paradoxical results described here. Our data shows that DcR3 can either increase resistance or enhance the cytotoxic effects of platinum and we theorize that the effects on platinum response may depend on which HSPGs are expressed on the cell surface. In SKOV-3 and OVCAR-3 cells the predominant HSPG is Syndecan-2. In these cells DcR3 exposure results in increased resistance to platinum. Conversely CaOV-3 cells express CD44v3 at more than 10 fold the level in the other cell lines and become more sensitive to lower doses of platinum after exposure to DcR3. In our original series of patient samples, DcR3 was identified in ascites fluid of all women with advanced EOC with a wide range of concentrations, 70–14,000 pg/ml and women with high DcR3 were twice as likely to manifest platinum resistant disease. Despite this statistically significant finding there were women with high levels who were platinum sensitive and women with lower levels who were platinum resistant (see Figure 1A). The cell line data presented here may offer an explanation for this in that cell lines with Syndecan predominant HSPG pattern (or perhaps, as described for survival in other cancers, cells that have lost CD44v3 expression) become more platinum resistant when chronically exposed to DcR3 while the cell line with CD44v3 as a more dominant HSPG become more platinum sensitive. Thus the effect of even very high levels of local DcR3 in terms of platinum responsiveness may depend on the cancer cells pattern of DcR3 binding partners and not just the presence of DcR3. We are currently collecting samples and clinical data on a larger group of women to verify this theory.

Neither Syndecan-2 nor CD44v3 have been extensively studied in EOC, however data from other tumor types is consistent with our preliminary findings and hypothesis. CD44 is a transmembrane molecule that functions as both an adhesion molecule and in some cases as a cell signaling receptor. The full molecule, often called CD44 standard (CD44S) has been found to be expressed or over expressed in a variety of cancers including EOC. In addition to CD44S, malignant cells can express a number of variant CD44 molecules as the result of post translational modifications. CD44v3 is one of these variants but is unique in that it is decorated with Heparan Sulfates, (i.e. it is a HSPG), as noted above. The role of CD44v3 in cancer is tumor type dependent; however in many malignancies studied, its expression is associated with a better prognosis compared to tumors that lack CD44v3 expression. In melanoma, CD44v3 expression is associated with other key prognostic factors resulting in a 75% 5 year survival in CD44v3 positive tumors vs. 45% in negative cases, *p* = 0.0072 [24]. Similarly, CD44v3 is present in the majority of benign uterine fibroids but is uniformly absent in uterine sarcomas [25]. In adenocarcinoma of the lung, CD44v3 is present in non-invasive lesions but is not expressed in the presence of frank invasion. In this tumor type, recurrence was more common and disease free survival was shorter in CD44v3 negative lesions in both univariate and multivariate analyses (*p* = 0.01 and 0.03 respectively) [26]. In squamous lesions of the uterine cervix CD44v3 is reduced in invasive vs. pre-invasive lesions; however in squamous vulvar lesions CD44v3 is increased in invasive lesions with a more aggressive natural history. Similarly in colon cancer CD44v3 expression is seen in over 50% of cases and was associated with a poor prognosis [27,28]. CD44v3 expression is variable and was not found to be related to prognosis in either direction in thyroid, gastric, gall bladder, or invasive transitional cell bladder cancers[29].

In EOC, CD44v3 has been seen in both tumors of low malignant potential and invasive disease. In invasive disease, lack of CD44v3 was associated with a poor prognosis. Lack of CD44v3 was more common in higher stage lesions and was associate with poor survival in both univariate (*p* = 0.027) and multivariate analysis (*p* = 0.026) independent of stage and lymph node status. These findings are in line with our hypothesis that CD44v3 expression is associated with a better response to platinum especially in the presence of high levels of DcR3[30].

Syndecan-2 is a HSPG that is known to function as a co-receptor for key growth factors including FGF-2, VEGF, EphB2, and TGFβ and to facilitate interactions between the extracellular matrix (ECM) and cell membrane/cell cytoskeletal functions such as vesicle transport. synaptic formation, and cell locomotion via filopodia formation[31]. Syndecan-2 is thought to be an important factor in normal development and its abnormal expression has been associated with colon and lung cancers, where it is found to facilitate metastasis by increasing motility and promoting angiogenesis [32,33]. Little is known about the functions of Syndecan-2 in EOC, although it is known to be expressed in both tumor associated stroma and on the surface of epithelial cells [34,35] . On the surface of cancer cells Syndecan-2 has been found to interact with other key cell surface signaling molecules including caveolin-2, RACK1, p120, and STAT3, all of which influence the activation of the oncogenes ras and Src, which in turn are key factors in the pathogenesis of EOC[36-40].

Although our data does not exclude the involvement of multiple mechanisms it suggests that DcR3 may alter response to platinum at least in part by regulating the expression of BRCA1. Our understanding of the functions of BRCA1 in ovarian cancer is evolving and complicated however there is consistent evidence of a role in response to DNA damaging agents such as platinum [41-43]. In 1998 Husain demonstrated that BRCA1 mRNA was upregulated and the protein overexpressed in platinum resistant sub-clones of both MCF-7 and SKOV-3 cells. In addition to platinum resistance BRCA1 overexpression was associated with improved DNA repair (an area we have not yet explored in our chronically exposed DcR3 system). In this study, transfection with BRCA1 anti-sense constructs was shown to reverse both processes [44]. Similarly, Horiuchi verified that suppression of BRCA1 lead to increased apoptosis in response to platinum and that this was associated with enhanced p53 and p21 function [45]. Our data are consistent with these findings in that DcR3 exposure results in increased BRCA1 expression in the two lines that became more resistant and reduced BRCA1 in the CaOV3 cells as they became more sensitive to platinum after chronic DcR3 exposure. In addition our array data support this by similar expression alterations of both p53 and p21 in OVCAR-3 cells and an increase an in p21 expression in CaOV3 cells (table 1). Further study into the potential role of BRCA1 in this process will require investigation into other possible mechanisms of BRCA1 regulation including assessment of its phosphorylation status and the physical location of the BRCA1 protein and or its protein complex partners in these cells.

## Conclusions

Based on the data presented we conclude that non-malignant cells contribute to the high levels of DcR3 found in the peritoneal cavity of women with ovarian cancer. DcR3 binds readily to EOC cells via HSPGs and alter their responsiveness to platinum chemotherapy. Two of three cell lines tested became more resistant to platinum were the third showed increased sensitivity to platinum. This paradoxical response appears to be related to expression pattern of HSPGs available on the cells surface to interact with. Although the mechanism behind this is not completely known alterations in DNA repair pathways including the expression of BRCA1 appear to be involved.

## Competing interests

The authors declare that they have no competing interest associated with this work.

## Authors’ contributions

JPC, MD. is the PI for this project and was responsible for the idea/hypothesis development and design of all of the experiments presented. He participated in the conduct of the experiments, analyzed and interpreted all of the data and wrote the manuscript. MF, BS is a senior research associate in Dr. JPC lab and performed the cell culture work, flow cytometry, MTT assays, and assisted in the analysis of these results. AK PhD is a research scientist in the Gynecologic Oncology division and performed the PCR array; RT-PCR and Western blot experiments. She participated in the analysis of these results and in compiling the data for manuscript preparation with Dr. JPC. NO, MD is a fellow in Gynecologic Oncology. She performed tissue culture and participated with the PCR array and analysis of these results. All authors read and approved the final manuscript.

## Pre-publication history

The pre-publication history for this paper can be accessed here:

http://www.biomedcentral.com/1471-2407/12/176/prepub
